# Patient-Reported Outcomes of Bicruciate Multiligament Versus Single Cruciate Multiligament Knee Injuries

**DOI:** 10.1177/03635465241293743

**Published:** 2025-01-01

**Authors:** Ingrid Trøan, Tone Bere, Inger Holm, Robert F. LaPrade, Lars Engebretsen, Gilbert Moatshe

**Affiliations:** * Orthopaedic Division, Oslo University Hospital, Oslo, Norway; † University of Oslo, Oslo, Norway; ‡ Twin Cities Orthopedics, Edina, Minnesota, USA; § Oslo Sports Trauma Research Center, Norwegian School of Sports Sciences, Oslo, Norway; ‖ Department of Experimental Orthopaedic Research, Institute for Surgical Research, Oslo University Hospital, Oslo, Norway; Investigation performed at Oslo University Hospital, Oslo, Norway

**Keywords:** multiligament knee injury, subjective outcome measure, bicruciate ligament, single cruciate ligament

## Abstract

**Background:**

Multiligament knee injuries (MLKIs) are heterogeneous, and bicruciate knee ligament injuries are considered a serious form of this injury. The current literature tends not to distinguish between single and bicruciate MLKI when reporting outcomes.

**Purpose:**

To investigate patient-reported outcomes after surgical treatment of MLKI comparing single cruciate MLKI with bicruciate MLKI. The secondary aim was to investigate the influence of different factors on patient-reported outcomes after surgery.

**Study Design:**

Cohort study; Level of evidence, 3.

**Methods:**

This study was designed as a cross-sectional cohort study. Patients who underwent surgical treatment for MLKI at a single level 1 trauma center between January 2013 and December 2020 were included in this study. Patient-reported outcomes included the Tegner score, Lysholm score, International Knee Documentation Committee (IKDC) subjective knee form, Knee injury and Osteoarthritis Outcome Survey (KOOS), and a visual analog scale for pain.

**Results:**

Of the 191 patients meeting the inclusion criteria, 124 (65%) agreed to participate and had a complete data set with a follow-up time at a mean 74 ± 27 months. Patients with single cruciate MLKI (type I) had significantly higher scores for IKDC (*P* = .007), Lysholm (*P* = .012), KOOS Pain (*P* = .04), KOOS Activities of Daily Living (*P* = .01), KOOS Sport and Recreation (*P* = .005), KOOS Quality of Life (*P* = .04), KOOS_4_ (which considers the subscales of Pain, Symptoms, Sport and Recreation, and Quality of Life) (*P* = .01), Tegner (*P* = .04), and visual analog scale for pain during activity (*P* = .004) when compared with patients with bicruciate MLKI (type II-type IV). Furthermore, age was significantly associated with a lower IKDC (*P* = .001), and an increased severity of injury was significantly associated with IKDC (*P* = .015), KOOS_4_ (*P* = .022), and Lysholm (*P* = .029) scores.

**Conclusion:**

MLKIs involving a single cruciate ligament had significantly higher patient-reported postoperative outcome measures compared with bicruciate MLKIs. Age and type of injury were important predictors for outcomes. Patients presenting with dislocated knees had lower patient-reported outcomes; however, there was no significant difference in outcomes between bicruciate MLKIs and patients presenting with dislocated knees.

Multiligament knee (MLK) injuries (MLKIs) are serious injuries that can have dramatic consequences for patients, and optimal management is still a challenge.^10,47,48^ Surgical management is considered the standard of care because nonoperative treatment has been reported to lead to inferior outcomes.^23,35,42,43,45,46^ These injuries are highly heterogeneous, and the severity is highly variable because of the mechanism of injury and the spectrum of concomitant injuries in addition to the ligament torn. Concomitant meniscal and cartilage injuries are common in MLKIs, reported in up to 55% and 48%, respectively.^#^Concomitant injuries will potentially influence the treatment choice and outcomes.

Bicruciate ligament injuries are more severe than those involving only 1 of the cruciate ligaments because they result in more significant instability and a higher risk of neurovascular injuries.^48^ Historically, bicruciate ligament injuries have been caused by high-energy injury mechanisms. However, recent data demonstrate that sporting injury mechanisms^7,24,29^ and ultra–low velocity mechanisms^1,41,49^ can cause these injuries as well. Ultra–low velocity injuries are usually seen in the morbidly obese and are associated with a high risk of neurovascular injuries and complication rates.^49^ In the literature, bicruciate MLKIs are also commonly termed knee dislocations (KDs) and are classified according to the knee ligament injury pattern and concomitant neurovascular or fracture injuries using the Schenck classification.^40^ However, recent literature has demonstrated the importance of documenting whether the knee was dislocated, because patients undergoing surgical management of a 3-ligament MLKI with true, documented KD had significantly worse clinical and functional outcomes than those with nondislocated knee joints.^17^

Even though bicruciate MLKIs are considered more serious injuries compared with MLKIs involving a single cruciate ligament, the current literature does not differentiate the 2 entities when reporting outcomes. In recent years, there has been a reported increase in classifying a single cruciate MLKI as KD I, causing some inconsistencies in reporting.^12^ Furthermore, the current clinical outcome studies are limited primarily to case series^8,15^ and the broad spectrum of injury severity^7,39^ makes it challenging to evaluate how the severity of injury and number of ligaments torn affects patient-reported outcomes.

Although a recent meta-analysis reported that patients with low-energy injuries had significantly improved postoperative Tegner activity scores compared with high-energy injuries,^6^ other studies^7,30^ reported no significant differences. Other factors that have been implicated to influence outcomes include age, concomitant cartilage injuries, and time from injury to surgery.

Therefore, the purpose of this study was to investigate outcomes after surgical treatment of MLKI comparing single cruciate MLKIs with bicruciate MLKIs. The secondary aim was to investigate the influence of meniscal and cartilage injuries, energy of trauma, age, and sex on patient-reported outcomes after surgery. The hypothesis was that patients with bicruciate injuries would have poorer outcomes than those with single cruciate ligament MLKIs.

## Methods

### Patient Population

**Figure 1.**
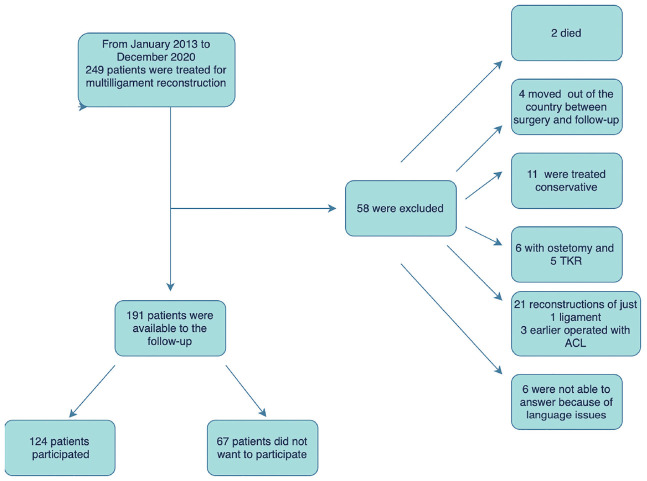
Flowchart of patients with multiple ligament knee injuries in the study. ACL, anterior cruciate ligament; TKA, total knee arthroplasty.

This was a cross-sectional cohort study and was approved by the ethical review board (REK Nord-IRB206446). All patients who underwent surgical treatment of an MLKI at the orthopaedic department at a single level 1 trauma center between January 2013 and December 2020 were identified for the study. Multiligament injuries were defined as an injury to ≥2 ligaments of the knee requiring surgical treatment, including anterior cruciate ligament (ACL), posterior cruciate ligament (PCL), medial collateral ligament (MCL), fibular collateral ligament (FCL), or posterolateral corner (PLC) with a minimum 24-month follow-up. Patients with concomitant intra-articular knee fractures requiring surgical treatment as well as those who underwent revision ligament reconstruction, tibial osteotomy, or total knee replacement (TKR) before the time of follow-up were excluded (Figure 1). The patients were contacted by phone and invited to participate in web-based questionnaires. Of the 191 patients meeting the inclusion criteria, 124 (65%) agreed to participate.

### Baseline Characteristics

Patient charts were retrospectively reviewed to obtain baseline characteristics. Age at the time of injury, sex, date of injury, side of injury, mode of injury, type of injury, meniscal and cartilage injuries, surgical procedures, treatment, and complications were obtained. A classification system derived from Poploski et al^34^ were utilized to classify the injury based on the pattern of MLKI: MLK 1–AM is ACL and MCL; MLK 1–AL is ACL and FCL; MLK 1–AML is ACL, MCL, and FCL; MLK 1–PM is PCL and MCL; MLK 1–PL is PCL and FCL; MLK 1–PML is PCL, MCL, and FCL; MLK 2 is ACL and PCL; MLK 3–M is ACL, PCL, and MCL; MLK 3–L is ACL, PCL, and FCL; and MLK 4 is ACL, PCL, MCL, and FCL. For the analysis, we grouped the patients into 5 groups that were used for this study:

Type I: ACL or PCL and MCL or FCL

Type II: ACL and PCL

Type III–L: ACL, PCL, and FCL

Type III–M: ACL, PCL, and MCL

Type IV: ACL, PCL, FCL, and MCL

Thus, type I were MLKIs involving a single cruciate ligament, while types II to IV were bicruciate MLKIs. Patients were grouped into high- and low-energy mechanisms of injury. A high-energy mechanism was defined as motor vehicle accidents, falls from heights >5 feet, and high-speed sporting activities such as downhill skiing. Low-energy mechanism included injury from ball sports, cross-country skiing, falls from heights <5 feet, or activities of daily living.^47^

### Patient-Reported Outcome Measures

The knee-specific patient-reported outcome measures (PROMs) included the Tegner score, Lysholm score, the International Knee Documentation Committee (IKDC) subjective knee form, and the Knee injury and Osteoarthritis Outcome Score (KOOS).^14,19,28,36-38,46^ The pain intensity of the injured knee at night, at rest, and in activity was measured on a 10-cm visual analog scale (VAS) with the endpoints of “no pain” and “maximum pain.”

### Surgical Treatment

Patients underwent surgery at a single center with different surgeons using anatomic reconstructions as described in the literature. The ACL was reconstructed using a bone–patellar tendon–bone or hamstring tendon autograft. All PCL tears had an anatomic double-bundle PCL reconstruction using Achilles and tibial tendon allograft, and the MCL was reconstructed using the LaPrade repair augmentation reconstruction technique.^27^ The LaPrade PLC reconstruction technique was utilized for PLC injuries.^25^

### Postoperative and Rehabilitation Protocol

In cases involving the PCL, patients were placed into a knee range of motion (ROM) brace immediately postoperatively, locked in full extension, with a transition to a dynamic PCL brace for 12 to 16 weeks once swelling decreased. Initially, the brace was set to 0° to 90°. In cases with no PCL injury, the patients used an ROM brace for 8 weeks with full ROM allowed. All patients practiced partial weightbearing with crutches and performed isometric quadriceps exercises, straight-leg raise exercises from the beginning, and more dynamic exercises when the ROM in the knee increased. After 8 weeks, patients shifted to full weightbearing and started strengthening exercises, including cycling. The PCL brace was discontinued at 12 to 16 weeks, and full active ROM exercises were continued. Patients were allowed to return to full activity when they achieved a minimum of 90% quadriceps muscle strength symmetry compared with the uninjured limb and had a full or near full knee ROM.

### Statistical Analysis

The groups were compared using the *t* test for continuous variables and the Wilcoxon rank-sum (Mann-Whitney) test for nonparametric, continuous variables. Multivariate influence on outcome variables was tested by regression analysis. A *P* value of .05 was considered statistically significant, and all tests were 2-sided. Patient characteristics and surgical details were presented as means and standard deviations for continuous variables and frequencies and percentages for categorical variables. Analyses and tests were performed using Stata SE 18 (Stata Corp LLC).

## Results

### Patient Characteristics

All 124 patients included had a complete data set; 45% of the patients were female and 55% were male. The mean age at the time of injury was 37 ± 13.7 years and the mean follow-up was 74 ± 27.3 months. In total, 25% and 75% were high-energy and low-energy injuries, respectively. A total of 64% were sports-related injuries, and skiing-related activities accounted for 34% of all cases (Table 1). The most frequent injury pattern was a multiligament injury involving a single cruciate ligament (type I; 52%), while 48% had bicruciate injuries (type II-type IV). All patients were admitted to the hospital after the surgery with a mean hospital stay of 3.4 ± 1.8 days. Concomitant chondral injuries and meniscal tears were found in 36% and 32% of the knees (Table 2), respectively, whereas 11% of the meniscal tears were meniscus root tears. Concurrent meniscal suture repairs and resections were performed in 30% and 5% of cases, respectively. Peroneal nerve injury was found in 11 patients (9%); 8 patients later underwent a tibialis posterior tendon transposition for treatment of their foot drop. No patients sustained a vascular injury. Overall, 13 patients (10%) developed joint stiffness, requiring a manipulation under anesthesia (MUA) to improve ROM at 4.5 ± 3.5 months after surgery. Five patients had an MUA alone, while 8 patients had an MUA and a concurrent lysis of adhesions. Twelve of the 13 patients had multiligament injuries involving the medial side, the majority of which (9 of 13) were 3-ligament injuries, with 7 of the 13 MLK 3-M injuries. Thus, knee ligament severity was associated with increased risk of joint stiffness requiring MUA. The mean age of those patients who did not want to participate was 31 ± 12 years, with 69% being male. The percentage distribution of the different types of injury, concomitant injuries, and chronicity is reported in Table 3. There was a significant difference in age between the responders and nonresponders for inclusion in the study, but there was no significant difference in gender, injury type, and concomitant injuries.

**Table 1:** Patient Characteristics and Injury Mechanism for Multiple Ligament Knee Injuries (N = 124)^*a*^

Age at injury, mean (SD)	37 (13.7)
Follow-up time, mo, mean (SD)	74 (27.3)
Sex (male/female), n (%)	68 (55) / 56 (45)
BMI, mean (SD)	27 (4)
Energy (high/low), n (%)	32 (25) / 93 (75)
Injury mechanism, n (%)	
Motor vehicle accident	1 (1)
Motorcycle	10 (8)
Ball sports	18 (15)
Skiing sports	42 (34)
Other sports	19 (15)
Other^*b*^	34 (27)

a BMI, body mass index.

b Injury mechanisms classified as “other” included falling on a slippery floor, falling from a height, getting run over by a dog, tripping over one's legs, and falling.

**Table 2:** Injuries According to Multiligament Knee Injury Classification, Associated Injuries, and Postoperative Complications for Responders^*a*^

Multiligament Injury Pattern	n (%)	Meniscal Injury, n	Cartilage Injury, n	Nerve Injury, n	Knee Dislocation, n	Knee Stiffness, n	Length of Hospital Stay in Days, mean (SD)	Acute Injury, <3 wk, n	Chronic Injury, >3 wk, n
MLK 1-AM	27 (22)	13	9	1	0	2	2.6 (1.2)	15	12
MLK 1-AL	27 (22)	5	10	5	0	0	2.9 (1.3)	23	4
MLK 1-AML	00 (0)	0	0	0	0	0	0	0	0
MLK 1-PM	5 (4)	1	0	0	0	1	4.2 (2.7)	2	3
MLK 1-PL	4 (3)	2	3	0	0	0	3.2 (0.5)	0	4
MLK 1-PML	1 (1)	0	1	0	0	0	4 (0)	0	1
MLK 2	13 (10)	7	4	1	1	0	3.3 (1.2)	9	4
MLK 3-M	34 (27)	9	15	0	9	7	4.1 (1.6)	28	6
MLK 3-L	11 (9)	2	3	4	6	2	3.9 (2)	8	3
MLK 4	2 (2)	1	0	0	2	1	4.5 (0.7)	2	0
Total, n (%)	124 (100)	40 (32)	45 (36)	11 (8)	18 (14)	13 (10)	3.4 (±1.8)	87 (70)	37 (30)

a ACL, anterior cruciate ligament; FCL, fibular collateral ligament; MCL, medial collateral ligament; MLK, multiligament knee; PCL, posterior cruciate ligament; MLK1 AM = ACL and MCL, MLK1 AL = ACL and FCL, MLK1 AML = ACL, MCL, FCL, MLK1 PM = PCL and MCL, MLK 1-PL = PCL and FCL, MLK 1-PML = PCL, MCL, FCL, MLK 2 = ACL and PCL, MLK 3-M = ACL, PCL, MCL, MLK 3-L = ACL, PCL, FCL, MLK 4 = ACL, PCL, MCL, FCL

**Table 3:** Injuries According to Multiligament Knee Injury Classification, Associated Injuries, and Postoperative Complications for Nonresponders^*a*^

Multiligament Injury Pattern	n (%)	Meniscal Injury, n	Cartilage Injury, n	Nerve Injury, n	Knee Dislocation, n	Knee Stiffness, n	Chronic Injury, n
MLK 1–AM	17 (25.4)	5	4	1	0	2	7
MLK 1–AL	17 (25.4)	5	3	3	0	1	5
MLK 1–AML	0	0	0	0	0	0	0
MLK 1–PM	1 (1.5)	1	0	0	0	1	0
MLK 1–PL	5 (7.5)	2	3	1	0	0	2
MLK 1–PML	1 (1.5)	0	0	0	0	0	0
MLK 2	6 (9.0)	1	1	0	1	0	5
MLK 3–M	13 (19.4)	2	4	1	3	1	5
MLK 3–L	5 (7.5)	2	0	1	2	0	0
MLK 4	2 (3.0)	0	0	0	1	0	0
Total, n (%)	67 (100)	18 (27)	15 (22)	7 (10)	7 (10)	5 (7)	24 (36)

a ACL, anterior cruciate ligament; FCL, fibular collateral ligament; MCL, medial collateral ligament; MLK, multiligament knee; MLKI, multiligament knee injury; PCL, posterior cruciate ligament; MLK 1–AL, ACL and FCL; MLK 1–AM, ACL and MCL; MLK 1–AML, ACL, MCL, FCL; MLK 1–PL, PCL and FCL; MLK 1–PM, PCL and MCL; MLK 1–PML, PCL, MCL, FCL; MLK 2, ACL and PCL; MLK 3–L, ACL, PCL, FCL; MLK 3–M, ACL, PCL, MCL; MLK 4, ACL, PCL, MCL, FCL.

### Patient-Reported Outcomes

At follow-up, the mean ± SD Lysholm score was 71 ± 16; mean IKDC was 58 ± 11; mean KOOS Pain, 78 ± 18; KOOS Symptoms, 72 ± 19; KOOS Activities of Daily Living (ADL), 85 ± 18; KOOS Sport and Recreation (Sport/Rec), 51 ± 29; KOOS Quality of Life (QoL), 62 ± 24; KOOS_4_ (which considers the subscales of Pain, Symptoms, Sport/Rec, and QoL), 64 ± 20; and mean Tegner score was, 3 ± 2.2. Of the entire group, 19% had KOOS QoL <44, which has been reported to suggest poor knee function or subjective failure after ACL surgery.^11^

#### Single Cruciate Versus Bicruciate Ligament Injuries

Patients with type I injuries had significantly higher scores for the IKDC (*P* = .007), Lysholm (*P* = .01), KOOS Pain (*P* = .04), KOOS ADL (*P* = .01), KOOS Sport/Rec (*P* = .005), KOOS QoL (*P* = .04), KOOS_4_ (*P* = .01), Tegner at follow-up (*P* = .04), and VAS pain during activity (*P* = .004) when compared with patients with type II to type IV injuries (Table 4). There were no significant differences between the 2 groups for KOOS Symptoms (*P* = .06), VAS pain at night (*P* = .27), and VAS pain at rest (*P* = .07).

**Table 4:** Outcome Scores Comparing Type I and Type II to IV Multiple Ligament Knee Injuries^*a*^

Outcome Score	Type I, Mean (SD) (n = 64)	Type II-IV, Mean (SD) (n = 60)	*P* value
Lysholm	75 (13)	68 (17)	**.01**
IKDC	61 (10)	56 (10)	**.007**
KOOS Pain	82 (15)	75 (20)	**.04**
KOOS Symptoms	75 (17)	68 (19)	.06
KOOS ADL	89 (13)	81 (22)	**.01**
KOOS Sport/Rec	58 (27)	43 (30)	**.005**
KOOS QoL	67 (22)	58 (25)	**.04**
KOOS_4_	68 (19)	59 (21)	**.01**
Tegner follow-up	3.6 (2.2)	3 (2.2)	**.04**
VAS pain at night	1.6 (2.1)	2 (2.4)	.27
VAS pain at rest	1 (1.4)	1.7 (1.3)	.07
VAS pain during activity	2.2 (2.1)	3.4 (2.7)	**.004**

a ADL, Activities of Daily Living; IKDC, International Knee Documentation Committee subjective knee form; KOOS, Knee injury and Osteoarthritis Outcome Score; KOOS_4_, covering the KOOS subscales Pain, Symptoms, Sport/Rec, and QoL; QoL, Quality of Life; Sport/Rec, Sport and Recreation; VAS, visual analog scale. Bold signifies statistical significance.

#### Documented Knee Dislocation

When comparing the outcomes for patients with documented KD (both single cruciate and bicruciate MLKI) and with patients without documented KD, patients without a documented KD had significantly increased scores for IKDC, KOOS Sport/Rec, KOOS_4_, and Tegner at follow-up (Table 5). When comparing the outcome scores for patients with bicruciate injuries with or without dislocation, there was no significant difference for any of the outcome scores (Table 6).

**Table 5:** Outcome Scores for Patients With and Without Documented Knee Dislocation^*a*^

Outcome Score	Dislocated Knees, Mean (SD) n = 18	Nondislocated, Mean (SD) n = 106	*P* value
Lysholm	66 (20)	72 (15)	.41
IKDC	54 (6)	59 (11)	**.02**
KOOS Pain	74 (20)	79 (17)	.30
KOOS Symptoms	65 (22)	73 (18)	.16
KOOS ADL	80 (22)	86 (17)	.13
KOOS Sport/Rec	35 (27)	53 (29)	**.02**
KOOS QoL	53 (24)	64 (24)	.08
KOOS_4_	55 (22)	66 (20)	**.04**
Tegner follow-up	2.3 (1.3)	3.4 (2.3)	**.03**

a ADL, Activities of Daily Living; IKDC, International Knee Documentation Committee subjective knee form; KOOS, Knee injury and Osteoarthritis Outcome Score; KOOS_4_, covering the KOOS subscales Pain, Symptoms, Sport/Rec, and QoL; QoL, Quality of Life; Sport/Rec, Sport and Recreation. Bold signifies statistical significance.

**Table 6:** Outcome Scores for Patients With Bicruciate Knee Ligament Injuries With and Without Documented Knee Dislocation^*a*^

Outcome Score	Dislocated Knees, Mean (SD) n = 18	Bicruciate Without Dislocation, Mean (SD) n = 42	*P* value
Lysholm	66 (20)	68 (16)	.99
IKDC	54 (6)	56 (12)	.48
KOOS Pain	74 (20)	75 (20)	.75
KOOS Symptoms	65 (22)	69 (19)	.94
KOOS ADL	80 (22)	81 (22)	.63
KOOS Sport/Rec	35 (27)	46 (31)	.20
KOOS QoL	53 (24)	60 (26)	.36
KOOS_4_	55 (22)	61 (21)	.26
Tegner follow-up	2.3 (1.3)	3.2 (2.4)	.18

a ADL, Activities of Daily Living; IKDC, International Knee Documentation Committee subjective knee form, KOOS, Knee injury and Osteoarthritis Outcome Score; KOOS_4_, covering the KOOS subscales Pain, Symptoms, Sport/Rec, and Quality of Life; QoL, Quality of Life; Sport/Rec, Sport and Recreation.

#### Variables Influencing Patient-Reported Outcomes

Male patients had a significantly higher outcome for the Tegner activity scale at follow-up compared with their female counterparts: 3.6 ± 2.3 versus 2.8 ± 2 (*P* = .031). There was no significant difference between men and women regarding the other patient-reported outcomes measures. Patients with cartilage injuries had significantly lower scores on both the IKDC (*P* = .003) and Tegner (*P* = .031) compared with those without cartilage injuries at surgery. There were no significant differences in the outcome scores between high- versus low-energy trauma and the presence or absence of meniscal injuries for any of the questionnaires at follow-up.

#### Multivariate Correlations

**Figure 2.**
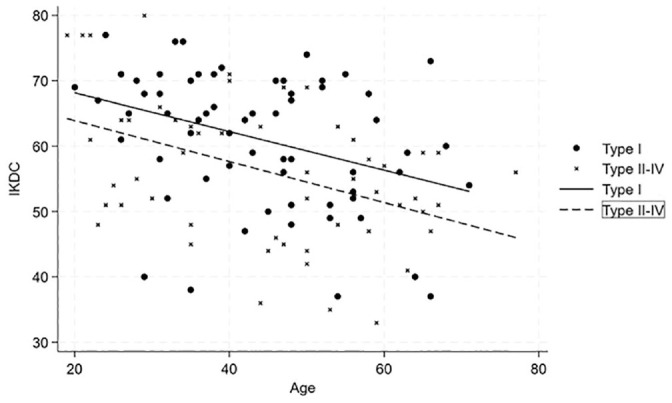
Scatterplot showing the relationship between the International Knee Documtation Comittee (IKDC) scores and the age for single cruciate injuries (circles) and bicruciate (X’s) multiligament injuries including regression lines for predicted values.

The adjusted *R*^2^ values from the multivariate regression analysis indicated that the model explained 23.9% of the variance in IKDC scores, 7.8% in KOOS_4_ scores, and 7.1% in Lysholm scores. The type of injury and age at follow-up were significant predictors for IKDC, with *P* values of .015 and .001, respectively. This indicates that increased age and more severe injuries had a negative linear influence on the IKDC score (Figure 2).

The severity of injury was also a significant predictor for KOOS_4_ (*P* = .022) and Lysholm (*P* = .029), implying that the most serious injuries lead to inferior scores. Factors such as sex, cartilage injury, the energy level of the trauma, and meniscal injuries were not significant predictors for any of the outcome scores.

## Discussion

The most important finding of this study was that patients with single cruciate MLKIs had significantly increased postoperative scores for IKDC, Lysholm, KOOS Pain, KOOS ADL, KOOS Sport/Rec, KOOS QoL, KOOS_4_, Tegner, and lower VAS pain during activity compared with those with bicruciate MLKIs at a mean 6 years after surgery. Age and knee ligament injury severity were the most important predictors for outcomes, with older patients and those with bicruciate ligament injuries having inferior outcomes. Nineteen percent of the patients had a KOOS QoL <44, which has been reported to be associated with poor knee function after ACL surgery,^36^ and 10% of all the patients had joint stiffness requiring an MUA.

This study demonstrated that single cruciate MLKIs are associated with improved knee function compared with bicruciate MLKIs at a mean 6 years after MLKI surgery. The study findings suggest that patients with single cruciate MLKIs might be expected to have superior outcome scores compared with those with bicruciate MLKIs. The findings were statistically significant; however, they might not be clinically significant considering that the differences between the groups were less than the minimal clinical important difference for the Lysholm, IKDC, and KOOS_4_ scores, which are 5.5 points, 11.5 points, and 8 points, respectively.^32,36^ Few studies have reported significant differences in the type of injury on subjective outcomes, partly because of small sample sizes, which makes it difficult to draw significant conclusions. Engebretsen et al^7^ categorized MLKI types differently when making statistical comparisons and reported that patients with MLK 4, injuries had significantly lower IKDC scores than those with MLK 2, MLK 3–M and MLK 3–M injuries. However, they considered the statistical comparison to be of limited value because of the small sample size of patients with KD IV injuries (n = 10). LaPrade et al^25^ found no significant differences in subjective outcomes between patients with injury to 1 cruciate ligament and those with bicruciate ligament injuries for sports-related MLKIs. Similarly, Borque et al^4^ found that patients with bicruciate injuries were slower to return to play after surgery but were able to return to a similar level compared with those with 1 cruciate injury. Their study did not report on any subjective outcome scores. Injuries involving both ACL and PCL reflect a higher energy trauma and a more complex injury pattern, leading to greater instability and often concomitant injury to the menisci and cartilage. Bicruciate ligament reconstructions are more complex, carry a higher risk of complication, and require longer, more intensive rehabilitation, heightening the risk of early osteoarthritis and instability.^30^ Differentiation based on the factors mentioned previously and the findings of this study should be considered.

When comparing the outcomes for patients with a documented KD (both single cruciate and bicruciate MLKIs) and for patients without a documented KD, patients without a documented KD had significantly higher scores for IKDC, KOOS Sport/Rec, KOOS_4_, and Tegner at follow-up. When comparing the outcome scores for patients with bicruciate injuries with or without a KD, there was no significant difference for any of the outcome scores. Only 2 patients with dislocated knees had a peroneal nerve injury and none had vascular injuries. This might suggest that outcomes after KDs without concomitant neurovascular injuries are comparable with those of bicruciate MLKIs without neurovascular injuries.

One of the most common postoperative complications after MLKI is stiffness. In this study, 92% of patients who developed stiffness requiring MUA had medial-based multiligament injuries, and 10 (77%) had bicruciate ligament injury. These findings are similar to those of the literature, showing that injury to the MCL increases the risk of stiffness.^22,25^ This could be explained by the higher incidence of such injuries. LaPrade et al,^25^ however, found no significant differences in the rate of arthrofibrosis between medial- and lateral-based multiligament injuries.

This study found that increased age was associated with lower IKDC scores. This finding is supported by the studies of Blokland et al^3^ and Levy et,^26^ which also found that young patient age was predictive of superior IKDC subjective outcome scores. A younger age likely reflects a higher activity level and a higher baseline functional status. Another possible explanation is that older patients tend to have more articular degeneration than younger patients. In the present study, we found that patients with cartilage injury at surgery had significantly lower scores on IKDC and Tegner compared with those without cartilage injuries at surgery. Those findings are supported by King et al^23^ and Moatshe et al,^30^ who reported that concomitant cartilage injuries were associated with poor IKDC scores. There was no significant difference between patients with meniscal injury and those without meniscal injury on the outcome scores. This could be because of the short follow-up time. One in 5 patients had a KOOS QoL <44, validated as a measure of inadequate knee function or graft failure after ACL reconstruction and represents a poor outcome after ACL surgery.^11^ Reports of subjective outcomes after multiligament knee reconstructions are limited in the literature. However, studies evaluating treatment failure depending on a KOOS QoL <44 after ACL reconstruction have reported that approximately 20% to 30% of patients after ACL reconstruction have treatment failure.^2,9,18^ A recent study from the Norwegian Knee Ligament Registry reported that 22% of patients stated subjective failure,^29^ which was associated with subsequent graft failure.^11^ In a recent study, Moatshe et al^31^ performed a study based on the Norwegian Knee Ligament Registry and reported that 49.5% and 46.5% of the patients had subjective failure (KOOS QoL <44) for isolated PCL reconstruction and combined PCL reconstruction, respectively, at 2-year follow-up. At 5-year follow-up, the subjective failure rates of isolated and combined PCL reconstruction were 46.7% and 34.2%, respectively, which was higher than in the present study.^31^

The present study found that men had a significantly higher Tegner activity scale than women, a finding not previously explored. This difference might be attributed to higher initial activity levels in male patients or general trends that men are more physically active in the Norwegian population, which persists with increasing age.^13^ A systematic review^5^ also found that women reported lower activity levels and knee-related outcomes after ACL injury and had lower odds of returning to sports. Sociocultural (eg, fear of injury, responsibilities related to work and family) and biological (eg, anatomy, hormones) factors were highlighted as a reason for the difference. Additionally, this study observed no significant difference in outcomes between high- and low-energy injury mechanisms after MLKI surgery. This finding contrasts with some reports,^28^ possibly because of the limited size of the high-energy group.

### Limitations

Limitations are acknowledged in this study. Nearly 35% of the eligible patients were lost to follow-up. One of the main limitations to this study was that there was no objective assessment such as clinical examination, imaging, and muscle strength testing. In addition, baseline PROMs were lacking, making it difficult to evaluate the improvement from baseline. Most patients (70%) were treated in the acute phase, where PROMs were poor; baseline PROMs would have improved the quality of the study. This is a single-center study in a relatively healthy population with few overweight patients, which can limit the external validity of this study. Another limitation is the lower percentage of high-energy injuries compared with other series, especially with very few motor vehicle–related mechanisms. Additionally, there was a lack of fracture dislocation patients, and no vascular injuries were observed, which further underscores the higher proportion of low-energy injuries in this cohort.

## Conclusion

Patients with MLKIs involving a single cruciate ligament had significantly higher PROMs compared with those with bicruciate MLKIs. Age and type of injury were important predictors of outcomes. Patients presenting with dislocated knees had lower patient-reported outcomes; however, there was no significant difference in outcomes between patients with bicruciate MLKIs and those presenting with dislocated knees.

## Notes

# References 15, 16, 20, 21, 23, 25, 33, 39, 44.
